# The experience of interval scans for adults living with primary malignant brain tumors

**DOI:** 10.1007/s00520-023-07818-z

**Published:** 2023-05-27

**Authors:** Florien W. Boele, Sarah E. Rudkin, Kate Absolom, Gary Latchford, Susan C. Short, Thomas C. Booth

**Affiliations:** 1 Leeds Institute of Medical Research at St James’s, St James’s University Hospital, University of Leeds, Leeds, UK; 2 Leeds Institute of Health Sciences, Faculty of Medicine and Health, University of Leeds, Leeds, UK; 3 School of Biomedical Engineering & Imaging Sciences, King’s College London, London, UK; 4 Kings College Hospital NHS Foundation Trust, London, UK

**Keywords:** MRI scans, Interval scans, Brain tumor, Patient experience, Qualitative interviews

## Abstract

**Purpose:**

People with primary malignant brain tumors (PMBT) undergo anti-tumor treatment and are followed up with MRI interval scans. There are potential burdens and benefits to interval scanning, yet high-quality evidence to suggest whether scans are beneficial or alter outcomes of importance for patients is lacking. We aimed to gain an in-depth understanding of how adults living with PMBTs experience and cope with interval scanning.

**Methods:**

Twelve patients diagnosed with WHO grade III or IV PMBT from two sites in the UK took part. Using a semi-structured interview guide, they were asked about their experiences of interval scans. A constructivist grounded theory approach was used to analyze data.

**Results:**

Although most participants found interval scans uncomfortable, they accepted that scans were something that they had to do and were using various coping methods to get through the MRI scan. All participants said that the wait between their scan and results was the most difficult part. Despite the difficulties they experienced, all participants said that they would rather have interval scans than wait for a change in their symptoms. Most of the time, scans provided relief, gave participants some certainty in an uncertain situation, and a short-term sense of control over their lives.

**Conclusion:**

The present study shows that interval scanning is important and highly valued by patients living with PMBT. Although interval scans are anxiety provoking, they appear to help people living with PMBT cope with the uncertainty of their condition.

## Introduction

People diagnosed with primary malignant brain tumors (PMBTs) face an unpredictable and complex illness, with high symptom burden and multiple episodes of invasive treatments [1]. Following initial treatment, MRI interval scanning is recommended as part of follow-up to detect progression or recurrence [2]. The recommended frequency of interval scanning varies depending on tumor type and specific guidelines, and may vary between country, for example, the US’s National Comprehensive Cancer Network recommending shorter intervals than the UK’s NICE guidelines [2, 3].

Interval scans are costly and time consuming for health services [2, 4], but may have benefits similar to monitoring biomarkers [5] including more available treatment options following early detection of progression. Scans can help in screening for late effects of treatment, and sequential imaging can be an important aspect of helping understand behavior and biology of recurrence after specific interventions. Scans may help inform patients and they may provide reassurance. However, regular scans without a change in symptoms may equally provoke anxiety and distress in patients, impacting quality of life [2, 4, 6, 7]. Pseudophenomena such as pseudoprogression can lead to further increased uncertainty and anxiety [8].

In other cancer populations, patient anxiety and fear of recurrence tend to rise with an upcoming interval scan, and then declines when uncertainty reduces following the disclosure of the imaging findings [9, 10]. In pediatric neuro-oncology, a qualitative study highlighted distress and worry about scans, but also feelings of relief, reassurance, and hope for the future following the results [11]. Waiting for results is consistently found to be difficult and anxiety provoking [9–11].

There is currently no research on the experience of interval scans for adults living with PMBTs, the optimal frequency of imaging, its economic gains or burdens, or the impact on quality of life and anxiety. There is uncertainty about whether follow-up alters outcomes of importance to patients [2, 4]. A recent position statement underscores the need for developing an evidence base around interval scanning practice in neuro-oncology [7]. The current study aims to add to this by gaining an in-depth understanding of how adults living with PMBTs experience and cope with interval scans.

## Materials and methods

### Study design

This was a qualitative study using in-depth semi-structured interviews following the constructivist grounded theory approach [12], conducted across two sites in the UK (Leeds Teaching Hospitals NHS Trust (LTHT) and Kings College Hospitals NHS Foundation Trust (KCH)). Patient representatives were involved in designing the research. The consolidated criteria for reporting qualitative research (COREQ) were used [13]. All quotes are pseudonymized. Ethical approval was obtained (Research Ethics Committee reference: 21/PR/0343).

### Participants

Adult (18 +) patients diagnosed with high-grade PMBT who were having interval scans (defined as “MRI scans at set intervals following completion of initial treatment”) who were proficient in English were recruited. Eligible patients were identified and approached by their clinical team before going through study information and consent procedures with the study coordinator (SER).

Using purposeful sampling, patients with experience of interval scanning were initially selected [14], then supplemented by theoretical sampling guided by constant comparative analysis, where analysis takes place concurrently with data collection [15, 16]. An example of theoretical sampling would be, that early interviews highlighted family support in aiding coping, so we sought out participants without strong family support to further develop coping categories. Recruitment continued until theoretical saturation was reached, meaning that no new insights emerged from additional interviews [14].

### Data collection

Interviews followed an interview guide (Table 1). All interviews were carried out remotely (via phone or Microsoft Teams), audio recorded, and transcribed verbatim. Participants were encouraged to interview alone but family caregivers could be present. Interviews were performed by a psychologist in clinical training (SER), with analysis supported by a team with extensive experience in research (KA, FWB, GL, TCB), neuro-oncology (FWB, TCB), and psychology (GL).

**Table 1:** Summary topic guide

Topic	Questions
Understanding of interval scans	*What were you told about…* • *Their purpose?* • *The scan itself and what it might be like?* • *Potential benefits and burdens?* • *Your choices when it comes to interval scans?* • *Receiving scan results?*
	*How was this information presented to you?*
Experience of interval scans	*What did you think/feel/do…* • *In the days leading up to the scan?* • *On the day of the scan?* • *After the scan, whilst waiting for your results?*
	*Was there any difference from one scan to the next? If so, what and why?*
Scan results	*What did you think/feel/do…* • *When you were told your scan results?* • *After you received your results?*
Benefits versus burdens	*Have interval scans been helpful for you, if so how?*
	*Have interval scans impacted on you or your everyday life in any way, if so, how?*
	*Would you choose to continue having interval scans, or prefer to scan following a change in symptoms?*

### Analysis

Constructivist grounded theory allows development of a theory or model with explanatory power, while reflecting on researchers’ own perspectives and interactions with the data [14–17]. Memos were used to record the research process, including decisions around analysis [16]. Coding followed three stages: initial, focused, and theoretical. Initial codes were generated for each participant while staying close to the data, which were then compared and refined before applying to the full dataset in focused coding. In theoretical coding the relationships between categories were defined and a theory developed [16]. Using models, revisiting memos, and reviewing the literature helped identify patterns and relationships between emerging categories. Modeling was used to provide visual representation of categories and their relationships [14, 16]. Through frequent team meetings (SER, FWB, KA, GL, TCB), consensus analysis was achieved.

## Results

### Participants

Interviews were conducted between July 2021 and February 2022. In total, 16 patients were approached and 12 participated. Four did not take part (two could not be contacted on the day of interview; one declined participation; one was unwell). Interviews lasted between 30 and 75 min (mean 50 min). In two cases, partners were present due to patients’ cognitive and language problems. Participant characteristics are displayed in Table 2.

**Table 2:** Participant sociodemographic and clinical characteristics

Participant pseudonym	Age group	Gender	Diagnosis (grade)	Treatments	Approximate years since diagnosis	Current scan interval (months)	Site
Anne	70–75	Female	GBM (4)	Standard	10	3	KCH
Julie	70–75	Female	GBM (4)	Standard plus additional	5	3–4	KCH
Ben	50–55	Male	GBM (4)	Standard plus additional	7	3	KCH
Sophie	50–55	Female	GBM (4)	TMZ and RT	4	3	KCH
James	50–55	Male	GBM (4)	Standard	< 1	3	LTHT
John	70–75	Male	GBM (4)	Standard	2	3	KCH
David	40–45	Male	GBM (4)	TMZ & RT	1	3	LTHT
Hannah	40–45	Female	GBM (4)	Standard	1	3	LTHT
Emma	45–50	Female	Solitary fibrous tumor of the dura (3)	Resection & RT	6	6	KCH
Adam	50–55	Male	Ependymoma (3)	Resection and RT	8	3–4	KCH
Jane	55–60	Female	GBM (4)	Standard	< 1	3	LTHT
Amy	40–45	Female	GBM (4)	Standard	< 1	3	LTHT

Standard treatment for glioblastoma (GBM) included surgical resection, radiotherapy (RT) plus concurrent temozolomide (TMZ) chemotherapy, followed by adjuvant TMZ. Treatments additional to standard of care included research trial-related

*GBM*, glioblastoma; *KCH*, Kings College Hospital NHS Foundation Trust; *LTHT*, Leeds Teaching Hospitals NHS Trust

### Grounded theory model

**Fig. 1:**
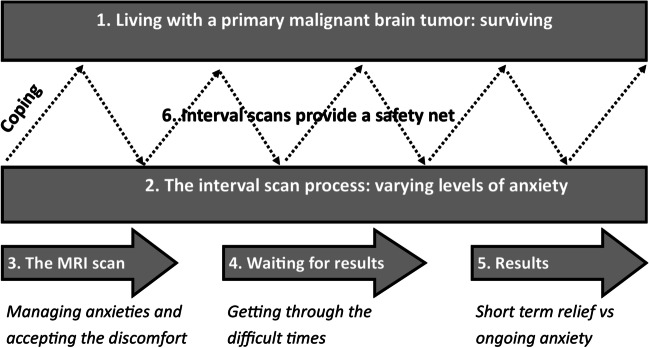
Grounded theory model

The grounded theory model resulting from the data is shown in Fig. 1, explaining how each of the categories are interlinked. Below, each category is described in more detail.

#### Living with a PMBT: surviving

Participants shared their experience of their diagnosis. They were all faced with a sudden and serious threat to their lives, accompanied by great uncertainty around prognosis and a feeling of loss of control. All felt they were doing what they could to prolong their life. Many participants talked about “having” to have treatments and scans, and felt that they had little choice:

*“I think it’s probably because, you know, it’s got to be done and you kind of just have to get on with it.” (Jane)*

All felt supported by family members or friends to some degree, which helped them cope. Some talked about past experience of trauma or illness—of themselves or family members—and how these had influenced their ability to cope now:

*“They didn’t think I’d make it, but I must have been quite tough…you know, I’ve been through quite big things in the past, so I know what to expect and just get on with it.” (John)*

#### The interval scan process: varying levels of anxiety

Participants’ experiences and access to support (as described in category 1) appeared to influence how they felt during the interval scan process. Before the scan, some worried about claustrophobia or having the MRI scan itself:

*“I’m still a little bit claustrophobic inside [those machines], and I have a few thoughts when I lie down that the machine’s going to stop working or it might crush me or something.” (Hannah)*

Prior to the scan, some were already worrying about what the results might show:

*“Doing the scan makes me think about the tumor, which slightly raises my anxiety levels about the possibility of return, which is always there.” (Adam)*

All but one participant, who had not yet received any results, reflected on the difficulty of waiting for scan results. For those who experienced worries pre-scan, the wait for results was described as even more anxiety provoking:

*“The most anxious period for me is in between the scan and getting the results; especially the lead up to getting the results because that’s, you know, I’ve had experience of that being a bad thing.” (Adam)*

Across the scan process (pre, during, post), participants often discussed the worry that the scan may confirm their biggest fear of disease progression. Some described experiencing hypervigilance to any sensations that might indicate progression, such as pain or headaches, even when they knew these could be normal:

“*Usually when I get up in a morning, I’m a little bit blurry. It takes me a little while to, um, sort of get with it during the day. But then the next morning after the scan I’ll get up I’ll be a bit blurry and think ‘Oh my god. The tumor must be growing’.” (Sophie)*

Participants described additional external stressors such as issues with arranging appointments, having their cannula fitted, with the MRI machine (e.g., it breaking down or having to attend mobile scanning units), and the impact of COVID-19 (e.g., travel to/from appointments; receiving results over the phone). Overall, there were individual differences in anxiety experienced by participants, which was impacted by both internal and external factors.

#### The MRI scan: managing anxieties and accepting the discomfort

Most participants stated that they did not find having a scan difficult, but rather described them as a “discomfort.” They referred to the conditions in the scanner as cold and noisy, that the machine was narrow and that they had to lie still for an extended period. Participants used different strategies to pass the time. Some simply rested, others described timing, counting, listening to the scanner’s noises, breathing, thinking about other things, or imagining being elsewhere:

*“I just shut my eyes and imagine that I'm on the beach. That’s always one of my coping mechanisms with claustrophobia.” (Sophie)*

Coping varied along with participants’ anxiety levels; some coped by comparing scans favorably with more invasive procedures or by making downward comparisons to other patients they considered less fortunate:

*“You’ve just got to man up; you have to get on with it. Some people are squeamish about having things going over you but none of that bothers me.” (John)*

*“I could imagine it could be difficult for anybody who hasn’t got anybody to take them. I’m lucky. My husband’s self-employed so he can always take time off and get me, get me there.” (Jane)*

Most talked about how small adaptions to the MRI scan environment could help (e.g., earplugs, music, a blanket), though not all adaptations were seen as helpful:

*“So, sometimes there’s a mirror that’s at an angle and you can see back to the room, and I don’t know if that’s a good thing or a bad thing! Because when I had the recurrence, I remember seeing them all crowding around you know the computer and I was, like, that doesn’t look good!” (Emma)*

Some participants described the experience of the scan as “isolated” and “lonely,” and talked about the importance of having staff around to support them through the scan:

*“Just the fact that you’re lying in a scanner that, you know you’re all on your own it’s quite isolating, it’s quite lonely. So, just for them to say you’re doing well, or you know we’ll be in in a minute; last ten minutes, you know just something encouraging to make you think, oh okay I'm doing all right.” (Amy)*

Overall, participants did not seem to find the MRI scan itself the most difficult part of the interval scan process.

#### Waiting for the results: getting through the difficult times

Waiting for results was the most difficult time for the majority of participants, associated with heightened anxiety, an increased sense of uncertainty, and lack of control. Participants described how they managed, often using emotion-focused and avoidance-related coping strategies such as distraction or trying not to think or talk about it:

*“I, you know try mentally try push those thoughts away because, and every reasonable person will be the same, like, I don’t know the result till I know the result. Stop trying to guess it.” (Sophie)*

*“I try not to expect anything and so when my girlfriend kinda says, you know, it’s probably going to be dreadful, and I’d stop her talking just so I'd rather not even think about it.” (David)*

Some looked for signs that their results might be positive:

*“You’re never gonna know until you get the results but like, I think also, I don’t know for sure, but I think if it is bad news...maybe they’d call me earlier.” (Ben)*

Others talked about their hope for good news; these were the participants who experienced less anxiety:

*“I just think, oh, what are they going to say. Has it grown back or is there, has it, is there any shrinkage; and then I think oh maybe they’re gonna tell me to go, I keep on imagining them saying oh, it’s completely gone.” (Hannah)*

Some acknowledged a lack of control over their illness and the scan results. They avoided being too hopeful, anxious, or trying to guess their results:

*“I try to think don’t worry about it because I can’t change anything; what will be will be.” (Jane)*

#### The results: short-term relief vs. ongoing anxiety

All but one participant had experience of receiving good results. Some participants described being at the peak of their anxiety at the point of being told, and being informed of good news provided a sense of relief:

*“When they say you are okay, and everything is going to be fine you think, ah, phew, that’s good, that’s a good thing.” (Anne)*

However, some were also aware that this relief was short lived, knowing they had to go through the same process again soon:

*“No, it’s good. Good. And I usually feel on top of the world. And then, you know, you do realise that you know you’re gonna have to go through it all again in three months’ time.” (Sophie)*

Some participants reported receiving indeterminate results, with ongoing anxiety until the scan was repeated:

*“That’s when they sent me to different department, a different scan machine cause they weren’t sure about something. I got a bit worried. I thought they’d found something, or, you know, it grew back or some-but it wasn’t that; they wanted to see something on a different machine.” (Hannah)*

A few participants had experience of bad news, with ongoing anxiety and loss of hope:

*“When it’s, you know, a bad result then the heightened-the anxiety stays and I ponder about things; start noticing it a bit more about what the possibilities and the negative, what negative outcomes can occur […] because until then you hope that it might have gone away permanently and you won’t have to worry about it again. But to be told that that hope has been dashed is deflating.” (Adam)*

#### Interval scans: provide a safety net

All participants found scans to be beneficial, and they all stated that they would rather have interval scans than wait for a change in symptoms. They described how having the scans made them feel “safer,” as they feared that without this monitoring, their illness would progress unnoticed, and then it would be “too late” to access further treatment. They also valued the ongoing connection to their medical team, which gave them a sense of security.

*“Peace of mind; just a bit of security really that someone’s there. They're gonna check things, not just leave me until I get symptoms” (Hannah)*

Some believed that having interval scans guided their treatment and helped them survive longer than they expected.

*“Well, I’ve been lucky that the tumor’s responded well to surgery each time and I’ve not headed into deterioration in quality of life because of it so again, that part of scanning helps with that because it enables them to get there. . .in time to ensure that I don’t get any negative effects. If it wasn’t for the scans, I’d probably be dead!” (Adam)*

Having interval scans allowed some participants to plan ahead, granting them some control over their lives.

*“I like to plan things and I like to be organised and I think once I know what, once I know again what I'm dealing with then I can, I can, take con-continue then.” (Amy)*

Interval scans provided a “safety net” for participants, helping them cope with the uncertainty of their illness.

## Discussion

In this multi-center qualitative study, we found that despite varying anxiety levels prior to, during, and after interval scans, adult patients with PMBTs found ways to cope with interval scanning. Scans helped manage the uncertainty of living with a PMBT, with reassurance offered with every clear result—however, short lasting. Participants also appreciated the ongoing connection to the treatment team and believed that interval scans would enable early detection and treatment of progression. This underscores the importance of instilling hope and having it protected by professionals [18, 19].

Varying levels of anxiety around having MRI scans and waiting for results have been reported in other patient populations [10, 11, 20, 21]. External stressors such as organizing appointments and technical scanner difficulties have also previously been found to heighten patients’ anxiety and discomfort [10, 22]. In the present study, a major component of anxiety was uncertainty around disease progression. This was also reported in long-term survivors of aggressive lymphoma, who reported fear of recurrence before CT surveillance scans [10]. Yet, unlike PMBT, lymphoma has a high cure rate and relapses are often detectable without scans—and lymphoma survivors said scans were inconvenient or that they felt “over tested,” something no one reported in the current study.

Most previous research on scan-related anxiety has focused on one-off scans that are not always cancer specific. Over time, our study participants adapted to MRI scans, normalizing the process and describing it as becoming routine. Similar experiences were reported in children with brain tumors and their parents [11, 23]. The wait for scan results being particularly difficult has also been noted in previous research [10, 11]. For context, at our recruitment sites scan results are typically given within 1 to 2 weeks. During this period of reduced sense of control and increased uncertainty, participants noted heightened anxiety and post-traumatic stress-related symptoms (e.g., hypervigilance, intrusive thoughts, and avoidance), similar to those seen early after a cancer diagnosis [24, 25].

To manage anxiety, our participants used various coping strategies, including avoidance, distraction, seeking support, making downward comparisons, hopeful thinking, problem solving, and emotional control. These strategies have also been reported by PMBT patients, in general [26–29]. Problem-focused coping strategies were used when the situation could be changed (e.g., strategies to reduce discomfort in the MRI scanner); and when there was little perceived control (e.g., waiting for results), more emotion-focused and avoidance-related coping strategies were reported. In adapting to scans over time, meaning-based coping strategies were used, with participants trying to adapt, control their situation, and increase their wellbeing. With the scan itself providing some sense of control [10, 11, 23], patients’ cognitive adaptation sometimes also included “illusions”—unrealistic positive beliefs aimed at increasing a person’s sense of control and wellbeing [30]. For some participants this took the form of a very optimistic outlook on the future. In general, interval scans helped participants to cope with the uncertainty of living with a PMBT.

This is the first study to look at how adults living with PMBTs experience and cope with interval scans, providing insight into their value to patients. Given the lack of previous research, a qualitative study using a rigorous grounded theory analysis was conducted to generate a model of patient experience. A study limitation is that different scan practices might impact on patient experiences differently. Although after adjuvant therapy for high-grade glioma, 81% of the UK’s 31 sites perform MRI at three monthly intervals, the length of follow-up is more variable [31]. Furthermore, there is variation in the timing and frequency of follow-up imaging during the adjuvant period. To mitigate potential sampling bias, patients were recruited from two sites. Another limitation is a potential selection bias, with those patients with progressive disease plausibly less likely to have been recruited. While half of our participants had experience of receiving bad news, the study does include a high proportion of longer-term survivors. Two participants with more cognitive issues were accompanied by a family member during their interview. Some cancer patients try to “stay strong” for family members, which may have influenced their responses [32]. Finally, this study was conducted during the Covid-19 pandemic which may have impacted on participant experiences and reflections (e.g., the pandemic being a time of increased uncertainty and stress; impact on travel; and communication methods of scan results) [33].

The value and benefit of neurooncological interval imaging in terms of clinical outcomes such as morbidity and overall survival remain unproven.^8^ Study participants, however, expressed no such doubt with a strong belief that interval scans would enable early detection and treatment. It is unknown how much this has been influenced by professionals instilling hope, or what impact more information on the limitations of interval imaging would have. It does appear that having interval scans has been incorporated into patients’ coping strategies, with the associated anxiety more than offset by the perceived reassurance that a good result offers, even if this is relatively short lasting. Future research should aim to expand the grounded theory model to include perspectives of patients with lower-grade PMBTs, as well as evaluating other strategies to help patients cope with living with a PMBT.

In conclusion, for high-grade PMBT patients, the benefits of interval scans outweighed the burdens. Interval scans provided them with a “safety net,” helping to reduce uncertainty, giving some sense of control and a connection to their medical team. Further understanding of the value of interval scans for adults living with PMBTs is likely to be determined by a combination of outcomes, including the clinical and economic gains or burdens.

## Data Availability

All raw data is held securely by the research team and access can be requested if required.
